# Impact of metagenomic sequencing on clinical outcomes in patients with suspected central nervous system infections: a retrospective case-control study

**DOI:** 10.3389/fcimb.2025.1677092

**Published:** 2025-11-07

**Authors:** Wenyan An, Yandong Zhang, Yong Liu, Tianshi Yang, Shiqi Bai, Peiwen Zhou, Junzhuo Si, Yuan Zhao, Yulu He, Yijia Pan, Yanfang Jiang

**Affiliations:** 1 Genetic Diagnosis Center, The First Hospital of Jilin University, Changchun, Jilin, China; 2 Department of Rheumatology and Immunology, The First Hospital of Jilin University, Changchun, Jilin, China

**Keywords:** central nervous system infections, mNGS, clinical outcome, antibiotic therapy, cerebrospinal fluid

## Abstract

**Objectives:**

Although the value of metagenomic sequencing (mNGS) in diagnosing pathogens in central nervous system infections (CNSi) has been confirmed, its impact on the clinical outcomes of patients remains to be elucidated. This study intended to investigate the clinical impact of cerebrospinal fluid (CSF) mNGS on the outcomes of patients with suspected CNSi.

**Methods:**

Between January 2022 and July 2024, patients who met both the inclusion and exclusion criteria were enrolled in the study and assigned to either the mNGS group (CSF tested by both mNGS and conventional microbiological tests [CMTs]) or the CMT group (CMTs alone). Following this, propensity score matching (PSM) was applied to balance baseline differences. The primary endpoint, time to clinical improvement, was then compared between the two groups and analyzed in stratified subgroups. Secondary endpoints included the rates of clinical improvement at 14 and 30 days, hospital stay, in-hospital mortality, and the proportion of GCS score <15.

**Results:**

A retrospective analysis of 338 patients was conducted, with 169 cases in each group. In the mNGS group, a comparison of diagnostic performance between the two testing methods demonstrated that mNGS yielded a significantly higher positivity rate in patients with CNSi compared to CMTs (67.5% vs. 18.3%, p < 0.001), identifying 111 pathogens in total, which was substantially more than the 24 detected by CMTs. Subsequent comparison of clinical outcomes between the groups showed that the duration until clinical improvement was significantly reduced in the mNGS group when compared to the CMT group (median: 14 days vs. 17 days; p=0.032). Moreover, a significantly higher percentage of patients in the mNGS group experienced clinical improvement within 14 days compared to those in the CMT group(42.6% vs. 31.4%; p=0.032). Subgroup analysis further revealed that the mNGS group’s superiority in clinical improvement over the CMT group was only evident in patients with CNSi, especially when complicated by pneumonia.

**Conclusion:**

The combination of mNGS with CMT significantly improves the clinical outcome of CNSi patients, offering greater clinical utility than traditional methods alone.

## 1 Introduction

Infectious diseases affecting the central nervous system (CNS), encompassing both the brain and spinal cord, represent a significant global health burden characterized by substantial pathogen diversity and high rates of morbidity and mortality. (Venkatesan et al., 2013) The profound impact of these infections is underscored by the estimation that meningitis alone contributed to approximately 32,000 deaths worldwide in 2016. (Yi et al., 2019) Timely identification of the causative pathogens and the precise administration of targeted antibiotic therapy are paramount for improving the prognosis of patients with CNS infections (CNSi). However, empirical antimicrobial therapy, often employed in the absence of rapid diagnostics, frequently results in suboptimal dosing or unnecessary broad-spectrum coverage, potentially compromising the patient’s prognosis.

Conventional microbial tests (CMTs), while foundational, have demonstrated limited diagnostic yield, identifying pathogens in less than half of suspected CNSi cases. (Leber et al., 2016; Glaser et al., 2006; Li et al., 2014) This critical limitation stems primarily from prolonged turnaround time and the inherent challenges associated with cultivating fastidious or slow-growing microorganisms *in vitro* (Liesman et al., 2017).

In recent years, metagenomic next-generation sequencing (mNGS) has evolved into a well-established technology that is widely applied in the comprehensive diagnosis of infectious diseases. By enabling unbiased detection of microbial nucleic acids directly from clinical specimens, mNGS provides broad-spectrum coverage of potential pathogens, including bacteria, viruses, fungi, and rare infectious agents. With simplified sample preparation and a significantly reduced turnaround time (typically 24–48 hours), mNGS enables rapid and comprehensive pathogen identification (Chiu and Miller, 2019; Han et al., 2019; Ramachandran and Wilson, 2020). Accumulating evidence over recent years has consistently demonstrated that the diagnostic sensitivity of mNGS for patients with CNSi significantly surpasses that of conventional culture-based methods, (Benoit et al., 2024; Wilson et al., 2019; Zhang et al., 2020) thereby offering the potential to revolutionize diagnostic decision-making (Martinez-Almoyna et al., 2019).

Nonetheless, while the analytical accuracy of mNGS for pathogen detection is increasingly well-established, robust evidence supporting its tangible impact on clinical management and, crucially, on improving patient outcomes remains comparatively limited. If mNGS fails to demonstrably influence therapeutic strategies or translate into enhanced prognoses, its widespread clinical utility may be constrained. Therefore, the primary objective of this study was to rigorously evaluate whether the clinical application of mNGS leads to improved outcomes for patients suffering from CNSi.

## 2 Methods

### 2.1 Patients and study design

A retrospective evaluation was conducted on patients with highly suspected CNSi who were admitted to the First Hospital of Jilin University between January 2022 and July 2024. CNSi was defined as the presence of both criteria (1) + (2) or (1) + (3): (1) Presence of ≥ 1 CNS-related symptom, including fever (body temperature > 38°C), headache, altered consciousness, or seizures, with or without accompanying nausea/vomiting, meningeal signs, or focal neurological deficits; (2) Laboratory findings: CSF analysis showing ≥ 1 marker of inflammation; (3) Neuroimaging evidence suggesting infection-related changes (MRI/CT). The exclusion criteria were as follows: (1) Patients with uncertain clinical diagnosis; (2) Patients with incomplete data and unclear case report; (3) Neonates; (4) Patients with traumatic brain injury; (5) Patients whose non-recovery or death was clinically judged to be primarily caused by a more severe underlying condition unrelated to CNS infection; Significant non-infectious factors potentially affecting the prognosis of CNSi patients were present, such as craniocerebral trauma or severe underlying diseases. (6) Patients who requested discharge during treatment. A total of 425 patients met the inclusion and exclusion criteria.

Non-central nervous system infections (non-CNSi) are defined as conditions affecting the brain, meninges, or spinal cord that present with clinical features similar to CNSi but lack microbiological evidence of infection, with the final etiology confirmed to be vascular, immune, metabolic/toxic, neoplastic, or other non-infectious mechanisms (Wang et al., 2024; Yuan et al., 2024).

Based on the method of CSF pathogen detection during clinical evaluation, all enrolled patients were stratified into two groups: the mNGS group (CSF tested concurrently by mNGS and CMTs) and the CMT group (CSF tested by CMTs alone). Propensity score matching was applied to balance potential confounders between groups. After matching, 169 patients were included in each group for the final analysis to evaluate the impact of mNGS versus CMTs on diagnostic performance and clinical management of CNSi.

### 2.2 Chart review and data collection

The electronic medical records of enrolled patients were retrospectively reviewed by experienced clinicians and clinical microbiology specialists. This comprehensive review included the initial and final diagnoses, the patients’ clinical presentations, therapeutic interventions, and therapeutic responses to antimicrobial therapy. General demographic information, laboratory results, and antimicrobial regimens of enrolled patients were systematically collected. It should be noted that during clinical practice, when patients exhibited clinical manifestations or routine CSF findings suggestive of infection, CSF samples were promptly collected for mNGS/CMTs to establish a definitive diagnosis and identify the causative pathogen. The majority of patients underwent mNGS within 1 to 3 days of admission. A subset of patients who developed hospital-acquired infections during treatment, primarily those with suspected postoperative neurosurgical infections, had samples collected during hospitalization. Overall, the median time from admission to mNGS sampling was 2 days (Interquartile range [IQR]:1-6.5), with a median reporting time of 1 day (IQR:1-2). Prior to the availability of mNGS or CMTs results, clinicians typically initiated empirical antimicrobial therapy based on initial clinical assessment. Once the pathogen was identified by mNGS or CMTs, the treatment regimen was adjusted accordingly for targeted therapy. Subsequently, medications were further modified dynamically based on the infection control status. For the adjustment of the antimicrobial regimen, it was categorized according to the following criteria: “Add,” indicating an escalation in the number or spectrum of agents; “De-escalation,” indicating a reduction in number or spectrum; and “Unchanged,” indicating no modification or changes inconsistent with pathogen profiles. These classifications adhered to the Antibiotic Stewardship Consensus (Moehring et al., 2021). The final results were all determined by the study team in consultation with the patient’s attending physician, or through consensus by intra-team discussion.

### 2.3 Clinical outcomes

The study baseline was defined as the time of the initial CSF sampling for mNGS or CMTs following a clinician’s strong suspicion of CNSi. The primary outcome was the time to clinical improvement, which was assessed based on previously published criteria and required all of the following to be met: (Zhao et al., 2023; Tunkel et al., 2017; Tängdén et al., 2011) (1) resolution of symptoms, including body temperature ≤ 37.3°C, recovery of Glasgow Coma Scale (GCS) score to baseline, and disappearance of headache and meningeal signs (without recurrence); (2) CSF parameters returning to normal or near normal; and (3) conversion to negative CMTs or mNGS results (if performed). In cases of ambiguity or disagreement, the final determination was made through attending physician consensus. For patients who died or failed to improve clinically, the adverse outcome was not attributed to CNSi if the following conditions were met at discharge: (1) continuous improvement in inflammatory markers, (2) resolution of meningeal symptoms, (3) negative CMTs or mNGS result (if tested), and (4) physician-judged presence of a more serious underlying cause accounting for treatment failure. Secondary outcomes encompassed the percentage of patients achieving clinical improvement at 14 and 30 days, the duration of hospital stays, hospital mortality rates, proportion of patients with GCS < 15, and the expenses associated with antimicrobial treatment.

### 2.4 Ethics statement

All procedures performed in studies involving human participants were conducted in accordance with national regulations, institutional policies, and the principles of the Declaration of Helsinki. The study protocol was approved by the Ethics Committee of the First Hospital of Jilin University (Approval No.AF-IRB-032-07).

### 2.5 Statistical analysis

To control for potential confounding between the mNGS and CMT groups, propensity score matching (PSM) was performed. A 1:1 nearest-neighbor matching algorithm was used with a caliper width of 0.2. The matching variables were selected to include key covariates that could potentially influence clinical outcomes, such as age, sex, comorbidities, and initial laboratory parameters (Disease events, including pneumonia, sepsis, and immunocompromised, occurred after the intervention, and their incidence did not differ significantly between the two groups, therefore, they were not included as matching variables to avoid selection bias). During the matching process, patients with extreme or unique baseline characteristics who could not find suitable matches within the caliper range—such as very advanced age or markedly abnormal CSF parameters—were excluded from the matched analysis. Covariate balance between groups was considered acceptable when the standardized mean differences (SMDs) for all variables were below 0.1 both before and after matching.

Continuous variables were expressed as medians along with IQR and assessed using the Mann-Whitney U test. Categorical variables were provided as counts (percentages) and evaluated with the χ² test or Fisher’s exact test. Furthermore, we assessed the interaction effects of mNGS with subgroup variables while controlling for covariates, and we presented the interaction through logistic regression models (p for interaction). For multiple comparisons, intergroup differences in continuous variables were first assessed using the Kruskal–Wallis nonparametric test, while categorical variables were analyzed with the χ² test. Variables demonstrating statistically significant differences (p < 0.05) were subsequently subjected to *post hoc* testing with Bonferroni correction to control the type I error rate. A p-value of less than 0.05 was deemed statistically significant. All statistical analyses and visualizations were conducted using SPSS (IBM, Chicago, IL), R Foundation for Statistical Computing, Vienna, Austria (version 4.4.2), and Origin 2024b.

## 3 Results

### 3.1 Patients

**Figure 1:**
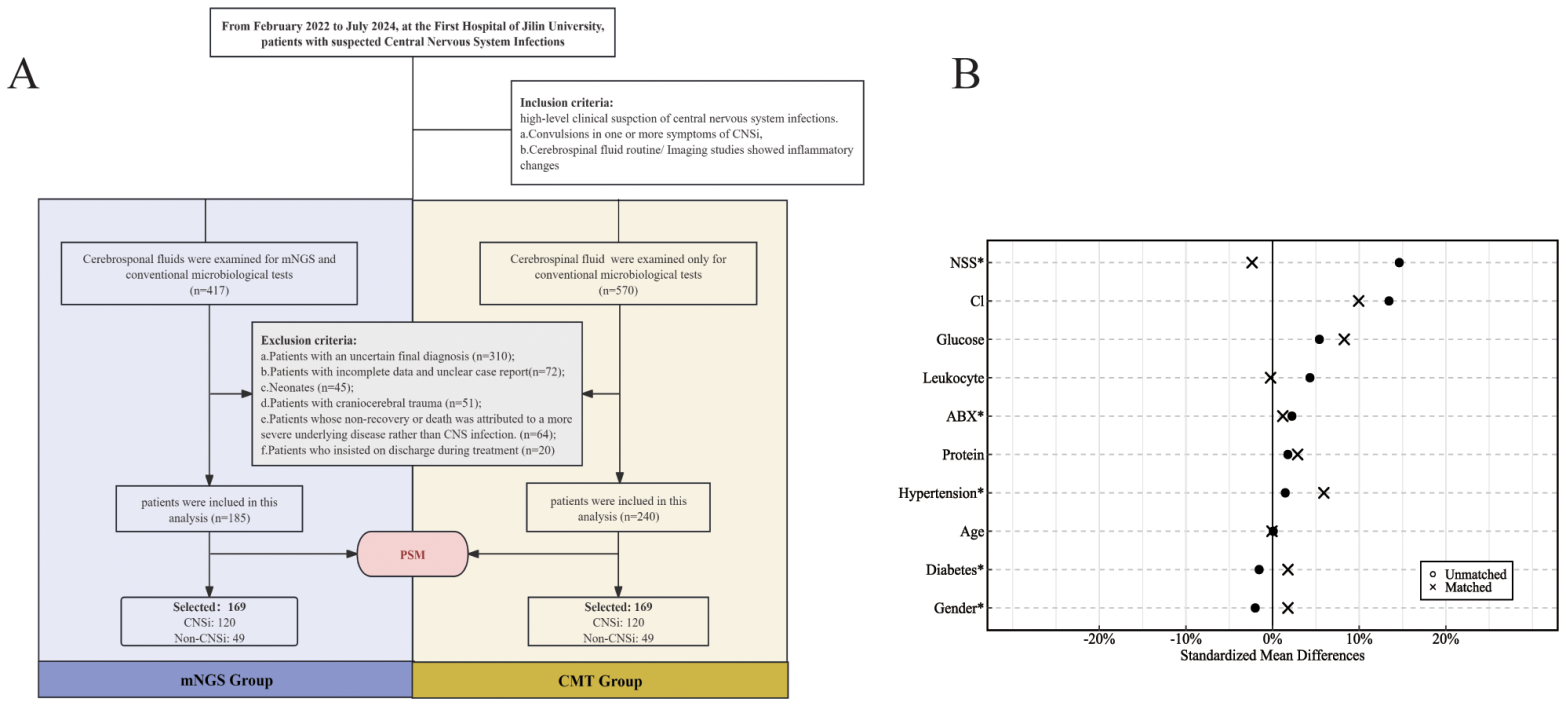
**(A)** Flowchart for enrollment; **(B)** Standardized bias plot of covariates before and after PSM (NSS, Neurosurgical surgery; ABX, Antibiotic exposure).

A total of 425 patients with suspected CNSi met the inclusion and exclusion criteria, including 185 in the mNGS group and 240 in the CMT group. After 1:1 PSM, a retrospective analysis was ultimately conducted on 338 patients (169 in each group) (**Figure 1A**). The matched mNGS group consisted of 120 patients with confirmed CNSi and 49 with non-CNSi diagnoses. Similarly, the matched CMT group included 120 CNSi and 49 non-CNSi cases. Non-CNSi diagnoses encompassed immune-mediated encephalopathies, malignant neoplasms, cerebrovascular disorders, metabolic and toxic encephalopathies (**Table 1**). Following PSM, all covariates achieved SMDs within the threshold of ±10%, indicating adequate balance in baseline characteristics between groups (**Figure 1B**; **Table 1**). The enrolled patients presented with an acute onset (**Table 1**). In the mNGS group, the median time from disease onset to specimen submission was 6 days (IQR, 3-13.5), with fever being the most common clinical symptom (71.0%). In the CMT group, the median time was also 6 days (IQR, 3-11). In addition, we compared the baseline characteristics of patients according to different pathogen types. The results revealed that eleven baseline characteristics differed significantly among patients with bacterial, viral, mixed, and unknown-pathogen infections (**Supplementary Table 1**). Further *post hoc* analyses demonstrated that CSF white blood cell count, glucose level, and protein concentration were significantly different between the bacterial and viral groups (p < 0.001). The combined use of these three parameters effectively distinguishes bacterial from viral infections. The mixed-infection group exhibited the most severe clinical manifestations, including a 35% incidence of sepsis and 65% incidence of electrolyte disturbances (**Supplementary Table 2**).

**Table 1:** Baseline characteristics.

Disease distribution	mNGS (n=169)	CMT (n=169)	*P* value
CNSi, n (%)	120.0 (71.0%)	120.0 (71.0%)	1.000
non-CNSi, n (%)	49.0 (29.0%)	49.0 (29.0%)	1.000
Immune-mediated	33.0 (19.5%)	22.0 (13.0%)	
Cerebrovascular disorders	2.0 (1.2%)	9.0 (5.3%)	
Metabolic encephalopathy	1.0 (0.6%)	5.0 (3.0%)	
Toxic encephalopathy	2.0 (1.6%)	8.0 (4.7%)	
malignant neoplasms	11.0 (6.5%)	5.0 (3.0%)	
Demographics			
Age,median (IQR)	37.0 (11.5-60.5)	43.0 (11.0-58.0)	0.717
Male, n (%)	94.0 (55.6%)	97.0 (57.4%)	0.742
Onset time	6.0 (3.0-13.5)	6.0 (3.0-11.0)	0.387
Clinical symptoms, n (%)			
Fever	120.0 (71.0%)	126 (74.6%)	0.463
Headache	95.0 (56.2%)	83.0 (49.1%)	0.191
Vomiting	47.0 (27.8%)	53.0 (31.4%)	0.475
Seizures	44.0 (26.0%)	41.0 (24.3%)	0.707
Altered consciousness	62.0 (36.7%)	75.0 (44.4%)	0.150
Underlying, n (%)			
Electrolyte	79.0 (46.7%)	71.0 (42.0%)	0.381
Hypoalbuminemia	41.0 (24.3%)	44.0 (26.0%)	0.707
Hypertension	37.0 (21.9%)	47.0 (27.8%)	0.208
Diabetes	22.0 (13.0%)	25.0 (14.8%)	0.637
Risk factors, n (%)			
Pneumonia	81.0 (47.9%)	77.0 (45.6%)	0.663
Sepsis	24.0 (14.2%)	18.0 (10.7%)	0.323
Immunocompromised	44.0 (26.0%)	43.0 (25.4%)	0.901
Diabetes	22.0 (13.0%)	25.0 (14.8%)	
Organ transplantation	5.0 (3.0%)	2.0 (1.2%)	
Chemotherapy	2.0 (1.2%)	2.0 (1.2%)	
HIV	2.0 (1.2%)	0 (0%)	
Immunosuppressive therapy	13.0 (7.7%)	14.0 (8.3%)	
Antibiotic exposure	117.0 (69.2%)	119.0 (70.4%)	0.813
Invasive surgery, n (%)			
Invasive mechanical ventilation	20.0 (11.8%)	25.0 (14.8%)	0.423
Neurosurgical surgery	40.0 (23.7%)	36.0 (21.3%)	0.602
Laboratory examination, median (IQR)			
CSF WBC, ×10^6^/L	79.0 (17.0-520.5)	126.0 (22.5-764.0)	0.346
CSF gulcose, mmol/L	3.2 (2.2-4.1)	3.3 (2.3-4.4)	0.610
CSF protein, g/L	1.0 (0.5-1.8)	0.9 (0.5-1.8)	0.767
CSF chloride, mmol/L	123.0 (117.8-126.6)	123.1 (119.5-126.3)	0.411

**Figure 2:**
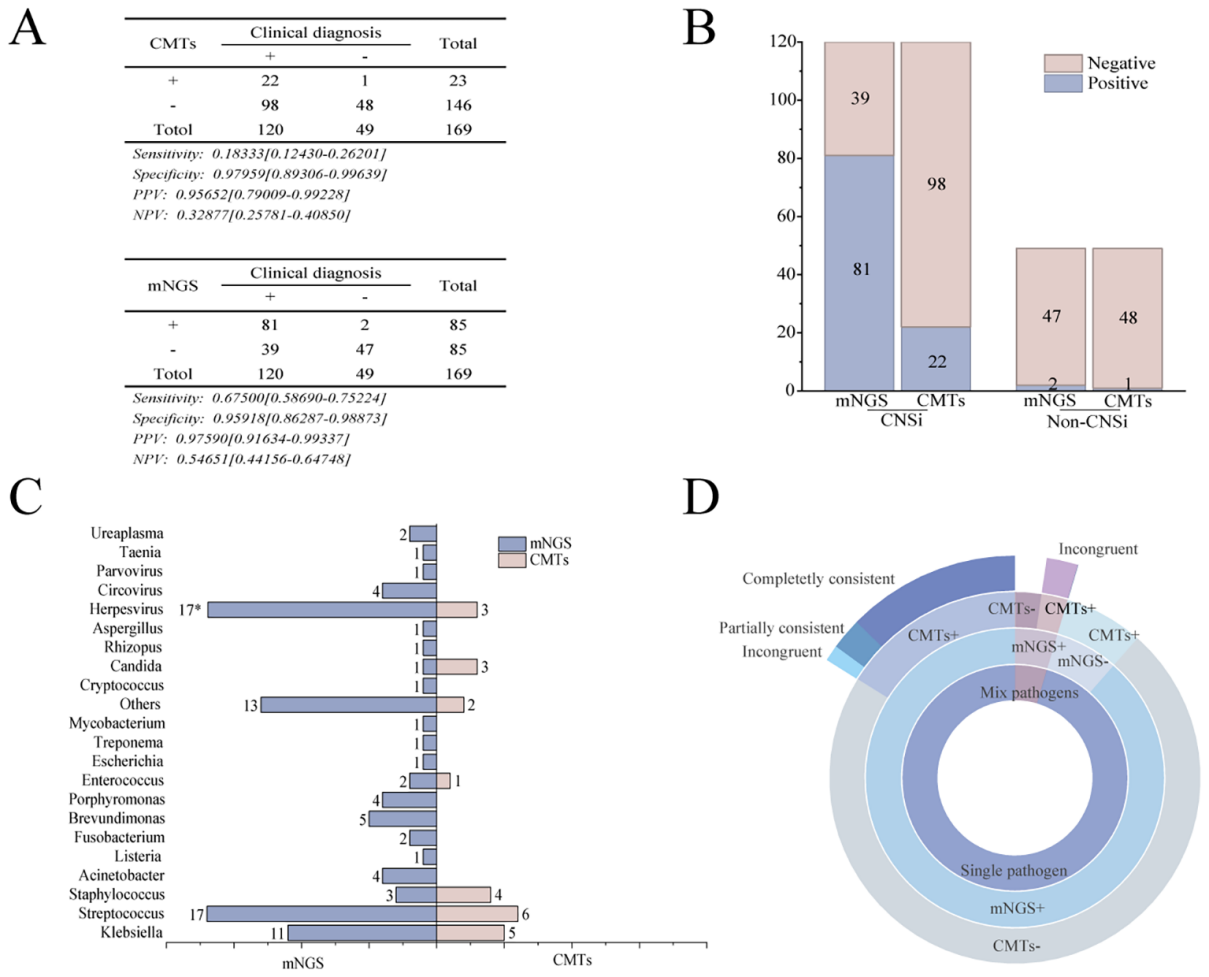
Comparison of pathogen identification capabilities between the mNGS and the CMTs. **(A)** Diagnostic performance of mNGS versus CMTs in the mNGS group:Sensitivity, specificity, positive predictive value (PPV), and negative predictive value (NPV); **(B)** Comparison of positive detection rates between mNGS and CMTs; **(C)** Pathogens detected at the genus level by mNGS and CMTs in CNSi patients (Use an asterisk (*) to indicate values greater than 17); **(D)** Concordance of pathogen detection between mNGS and CMTs.

Within the mNGS group, comparative analysis of the two detection methods showed that mNGS outperformed CMTs in sensitivity, positive predictive value, and negative predictive value (**Figure 2A**). Among the 120 patients in the mNGS group ultimately diagnosed with CNSi, mNGS failed to detect 39 cases, significantly fewer than the 98 cases missed by CMTs (p < 0.001, **Figure 2B**). Moreover, mNGS correctly identified 65 cases that tested negative by CMTs. In both methods, bacterial pathogens were the most frequently detected, with *Klebsiella* spp. and *Streptococcus* spp. representing the predominant genera responsible for bacterial meningitis. Overall, mNGS identified a total of 111 pathogens, primarily bacteria (58.6%), followed by viruses (35.1%) and fungi (3.6%). In contrast, CMTs detected only 24 pathogens in total (**Figure 2C**). In addition, concordance between the two methods was limited in cases of monomicrobial infection: only 11 cases showed complete agreement when both methods were positive, one case showed partially consistent, and two cases showed incongruent. Notably, no concordance was observed in cases of mixed infections (**Figure 2D**).

**Figure 3:**
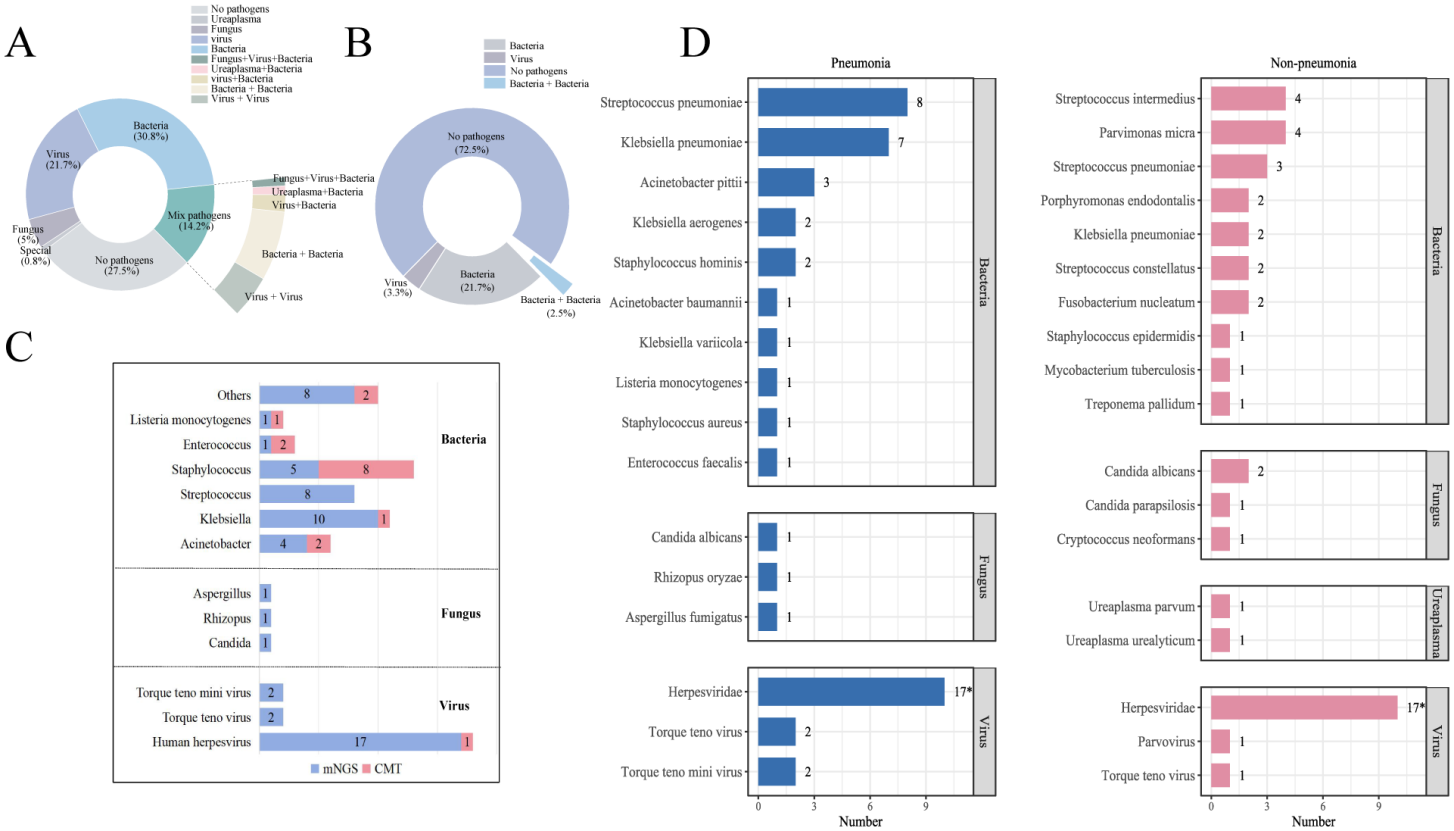
**(A, B)** Summary of CSF pathogen testing results from CNSi cases between mNGS and CMT groups; **(C)** Pathogen profiles in patients with CNSi complicated by pneumonia, in the mNGS and CMT groups. **(D)** Top 10 pathogen profiles in CNSi patients with in the mNGS group (Use an asterisk[*]to indicate values greater than 10).

Regarding pathogen detection performance, the mNGS group had a significantly higher positivity rate compared to the CMT group (72.5% vs. 27.5%, p<0.001; **Figures 3A, B**). Notably, the mNGS group demonstrated a distinct advantage in identifying viral infections and mixed-pathogen infections. A total of 26 cases of viral infections (21.7%) were detected by the mNGS group, compared to only four case (3.3%) identified by the CMT group. And all enrolled patients in both groups received empirical antimicrobial therapy. However, a significantly higher proportion of patients in the mNGS group had their treatment regimens optimized based on pathogen detection results (24.3% [41/169, mNGS] vs. 10.7% [18/169, CMT]; p<0.001; **Table 2**). Among patients with CNSi, particularly those complicated by pneumonia, mNGS facilitated more accurate pathogen identification and guided targeted antibiotic adjustment (**Figure 3C**, **Table 2**). The pathogen spectrum in the mNGS group demonstrated that CSF samples from CNSi patients with concurrent pneumonia more frequently yielded respiratory pathogens compared to those without pneumonia (**Figure 3D**). Furthermore, the time to treatment adjustment in the mNGS group was significantly shorter than that in the CMT group (3.0 [2.0–3.5] vs. 3.5 [3.0–5.0]; p = 0.015; **Table 2**). This difference remained statistically significant among patients with CNS infections (3.0 [2.0–3.0] vs. 4.0 [3.0–5.0]; p = 0.010) as well as in those with CNS infections complicated by pneumonia (3.0 [2.0–3.0] vs. 4.0 [3.0–5.0]; p = 0.024).

**Table 2:** Change of antimicrobial stewardship.

Outcome	mNGS	CMT	*P* value
All patients	169	169	
Changed, %	41.0 (24.3%)	18.0 (10.7%)	<0.001
Changed-Time,median (IQR)	3.0 (2.0-3.5)	3.5 (3.0-5.0)	0.015
CNSi	120	120	
Changed, %	36.0 (30.0%)	15.0 (12.5%)	<0.001
Changed-Time,median (IQR)	3.0 (2.0-3.0)	4.0 (3.0-5.0)	0.010
CNSi with pneumonia	54	48	
Changed, %	19.0 (35.2%)	5.0 (10.4%)	0.003
Changed-Time,median (IQR)	3.0 (2.0-3.0)	4.0 (3.0-5.0)	0.024

### 3.2 Primary outcome

The median time to clinical improvement was significantly shorter in the mNGS group compared to the CMT group (14 d vs. 17 d; p = 0.032; **Table 3**). Subgroup analysis revealed that this difference was primarily driven by patients with CNSi, as a significant reduction in time to clinical improvement was observed in the mNGS group compared to the CMT group (13 d vs. 17 d; p = 0.029, **Table 4**), whereas no significant difference was observed among patients with non-CNSi (17 d vs. 17.5 d; p = 0.570; **Table 4**). Notably, further analysis demonstrated markedly shorter days required for clinical improvement among CNSi patients complicated by pneumonia in the mNGS group compared to that of the CMT group (12 d vs. 17.5 d; p = 0.012; **Table 4**). Among CNSi patients without pneumonia, no significant differences were observed in the time to clinical improvement between the two groups (15 d vs. 17 d; p = 0.403; **Table 4**).

**Table 3:** Outcomes in the clinical improvement.

Outcome	mNGS (169)	CMT (169)	*P v*alue
Primary outcome			
Time to clinical improvement, median (IQR)	14.0 (9.0-23.0)	17.0 (12.0-22.8)	0.032
Secondary outcomes			
Clinical improvement within 30 d, n (%)	123.0 (72.8%)	120.0 (71.0%)	0.717
Clinical improvement within 14 d, n (%)	72.0 (42.6%)	53.0 (31.4%)	0.032
Length of hospital stay, median (IQR)	22.0 (14.0-32.5)	22.0 (15.0-32.0)	0.992
Mortality during hospitalization, n (%)	3.0 (1.8%)	6.0 (3.6%)	0.311
GCS<15, n (%)	24.0 (14.2%)	25.0 (14.8%)	0.877

**Table 4:** Outcomes in CNSi and non-CNSi patients.

Outcome	mNGS (169)	CMT (169)	*P* value
CNSi	120	120	
Time to clinical improvement, median (IQR)	13.0 (9.0-22.0)	17.0 (12.0-22.3)	0.029
Clinical improvement within 30 d, n (%)	88.0 (73.3%)	86.0 (71.7%)	0.772
Clinical improvement within 14 d, n (%)	53.0 (44.2%)	36.0 (30.0%)	0.023
Length of hospital stay, median (IQR)	22.0 (14.0-31.0)	23.0 (14.3-31.8)	0.688
Pneumonia	54	48	
Time to clinical improvement, median (IQR)	12.0 (9.0-18.8)	17.5 (13.3-22.0)	0.012
Clinical improvement within 30 d, n (%)	37.0 (68.5%)	32.0 (66.7%)	0.842
Clinical improvement within 14 d, n (%)	25.0 (46.3%)	10.0 (20.8%)	0.007
Length of hospital stay, median (IQR)	23.0 (17.8-28.5)	25.0 (15.3-32.5)	0.692
Non-Pneumonia	66	72	
Time to clinical improvement, median (IQR)	15.0 (9.0-23.0)	17.0 (10.8-25.3)	0.403
Clinical improvement within 30 d, n (%)	51.0 (77.3%)	54.0 (75.0%)	0.755
Clinical improvement within 14 d, n (%)	28.0 (42.4%)	26.0 (36.1%)	0.448
Length of hospital stay, median (IQR)	21.0 (13.0-34.3)	22 (14.0-31.5)	0.780
Non-CNSi	49	49	
Time to clinical improvement, median (IQR)	17.0 (8.0-27.0)	17.5 (12.0-23.3)	0.570
Clinical improvement within 30 d, n (%)	35.0 (71.4%)	34 (69.4%)	0.825
Clinical improvement within 14 d, n (%)	19.0 (38.8%)	17.0 (34.7%)	0.675
Length of hospital stay, median (IQR)	25.0 (13.5-36.0)	21.0 (16.0-32.5)	0.606

### 3.3 Secondary outcome

**Figure 4:**
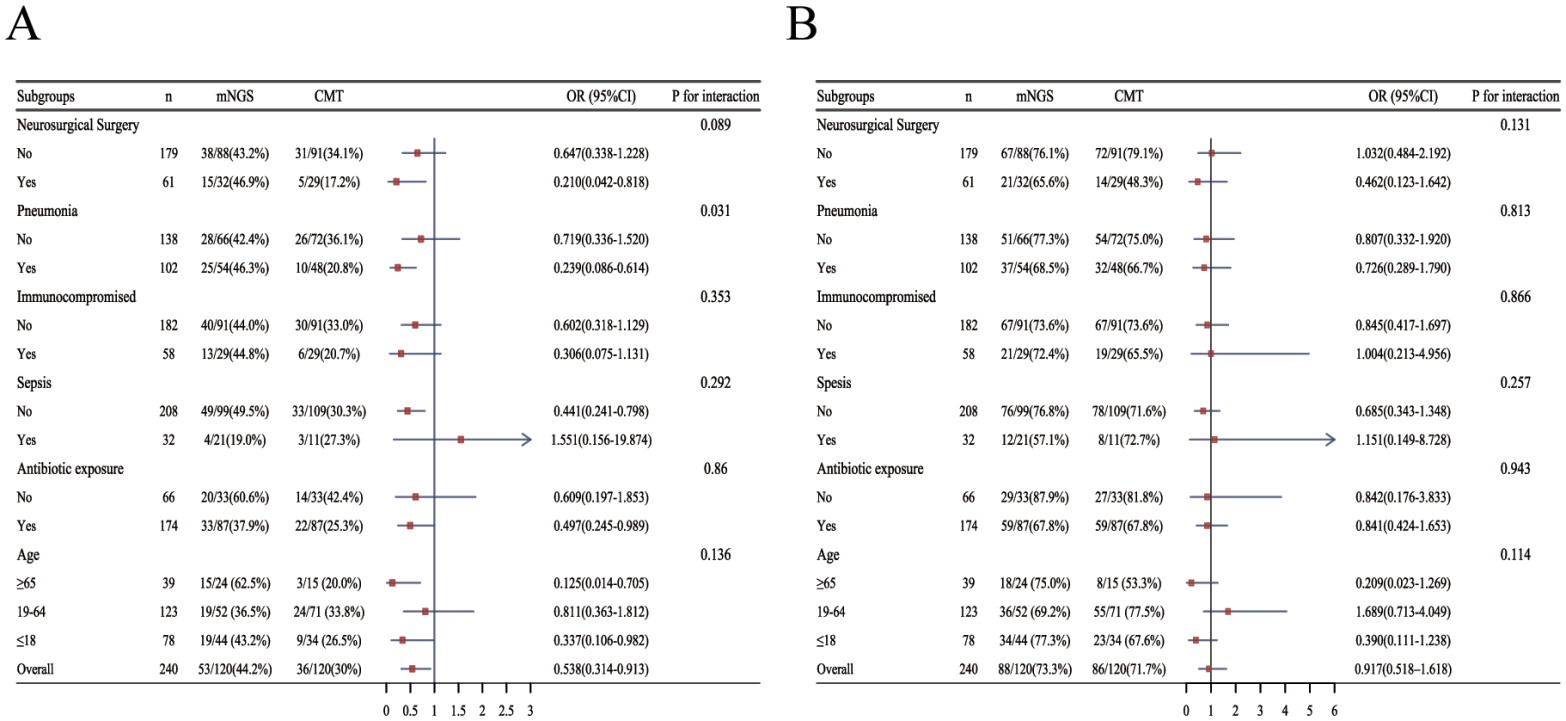
Forest plot of subgroup analysis for the proportion of clinical improvement within 14 days and 30days in patients with CNSi. (OR<1, the percentage of clinical improvement in the mNGS group was higher than that in the CMT group) **(A)** within 14 days; **(B)** within 30 days (Due to limited sample size, including age group and antibiotic exposure group as covariates for each other resulted in excessively wide confidence intervals, compromising the stability of the results. Therefore, in their respective subgroup analyses, these variables were not included as mutual covariates.).

Within 14 days, clinical improvement was achieved in 72 patients (42.6%) in the mNGS group, compared with 53 patients (31.4%) in the CMT group (p = 0.032; **Table 3**). CNSi patients showed similar disparities between the two groups, with the mNGS group showing a substantially higher proportion of clinical improvement within 14 days (44.2% [53/120, mNGS] vs. 30.0% [36/120, CMT]; p=0.023; **Table 4**). Subgroup analysis for the percentage of 14-day improvement among CNSi patients showed that mNGS conferred a significantly greater benefit in those with concurrent pneumonia (p=0.031; **Figure 4A**), with a significantly higher proportion of 14-day improvement in the mNGS group compared to the CMT group (46.3% [25/54, mNGS] vs. 20.8% [10/48, CMT]; p=0.007; **Table 4**). Among post-neurosurgical patients, the clinical improvement rate in the mNGS group was also markedly higher (46.9% vs. 17.2%), and a mild trend toward interaction was observed (p=0.089) (**Figure 4A**). Other subgroups (immunocompromised state, sepsis or not, history of antibiotic exposure or not, and age groups) did not demonstrate any statistically significant variations in the percentage of clinical improvements within 14 days between the two groups (**Figure 4A**).

There was no significant difference between the two groups in the proportion of patients achieving clinical improvement within 30 days (**Table 3**), nor in 30-day outcomes among patients with CNSi or those with CNSi complicated by pneumonia (**Table 4**). A subgroup analysis of 30-day improvement proportions is presented in **Figure 4B**, which also revealed no significant differences across subgroups.

None significant differences were obtained between the two groups regarding other secondary outcomes (**Table 3**), including length of hospital stay (p=0.992), mortality during hospitalization (p=0.311), and proportion of GCS<15 (p=0.877). Additionally, we compared laboratory parameters at the time of initial clinical suspicion of CNSi across different clinical outcomes. Statistically significant differences were observed in CSF protein (*p* = 0.008), and serum high-sensitivity C-reactive protein (CRP) levels (p=0.015, **Supplementary Figure 1**).

## 4 Discussion

While numerous studies have established the superior diagnostic accuracy and sensitivity of mNGS compared to CMTs for pathogen identification (Wilson et al., 2019; Zhang et al., 2020; Miller et al., 2019; Zhang et al., 2022), the critical question of whether this technological advancement translates into tangible clinical benefits remains inadequately addressed. Recent studies have begun to explore the role of mNGS in guiding clinical decision-making (Niles et al., 2020; Liu et al., 2024; Lai et al., 2025), however, systematic analyses assessing its clinical benefit in CNSi patients are still lacking. Li et al. reported that mNGS improved the precision of antibiotic use in patients with severe CNS infections, but the study was limited by a small sample size, absence of a conventional testing control group, and no observed improvement in clinical outcomes (Feng et al., 2023). To address this pivotal knowledge gap, we pioneeringly conducted a retrospective comparative analysis evaluating whether the integration of mNGS into diagnostic workflows enhances clinical outcomes in patients with suspected CNSi compared to CMTs alone. Our study demonstrates that mNGS holds significant clinical value in the management of patients with CNSi. Compared with CMTs alone, combined mNGS and CMTs not only substantially accelerates clinical improvement in CNSi patients but also more frequently guides the optimization and adjustment of antimicrobial therapy. It is important to note that the rapid turnaround time of mNGS constitutes a critical component of its diagnostic value and serves as a key mechanism for enabling earlier and more precise therapeutic interventions. Therefore, the observed differences in time to clinical improvement between the two groups should not be interpreted as methodological bias; rather, they genuinely reflect the clinical benefits of mNGS in facilitating earlier pathogen identification and more timely treatment adjustments in real-world practice. These findings indicate that the diagnostic advantage of mNGS extends beyond higher pathogen detection rates to tangible clinical benefits. Notably, this advantage is particularly pronounced in CNSi patients with concomitant pneumonia. Compared with patients without pneumonia, those with pneumonia exhibit a higher proportion of respiratory pathogens in the CSF, such as Klebsiella pneumoniae and Streptococcus pneumoniae. Moreover, in these patients, mNGS more frequently informs adjustments to antimicrobial therapy than CMTs, further highlighting its clinical utility in complex infectious scenarios. Overall, this study underscores the pivotal role of mNGS in the diagnosis and management of CNSi, providing robust evidence to support precision diagnostics and improved patient outcomes.

Our study employed “time to clinical improvement” as the primary outcome measure patient-centered endpoint that provides a more nuanced assessment of therapeutic efficacy than surrogate markers. We found that patients undergoing combined mNGS and CMTs experienced significantly accelerated clinical improvement compared to those receiving CMTs alone. Importantly, this benefit was observed exclusively in patients with confirmed CNSi, with no significant difference observed in non-CNSi cases. This finding is expected, given the superior pathogen detection capability of mNGS compared to CMTs. Consequently, the clinical advantage of mNGS versus CMTs in improving patient outcomes is likely to be confined to infectious diseases. Furthermore, this difference was limited to the 14-day period because the mNGS group experienced a considerably faster rate of improvement in clinical outcomes within that time frame than the CMT group. In contrast, no significant difference in the 30-day clinical improvement rate was observed between the two groups. The absence of significant differences in secondary outcomes such as the proportion of clinical improvement within 30 days, GCS scores, and in-hospital mortality may reflect both the limited enrollment of critically ill patients. Notably, although mNGS demonstrated a clear advantage over CMTs in terms of clinical improvement, it did not show a significant impact on mortality. This suggests that the benefits of pathogen-based diagnostics may not necessarily translate into short-term improvements in survival outcomes. Several factors may explain this. First, patients’ final outcomes are influenced by multiple factors, including disease severity and comorbidities, which may play a more decisive role during disease progression than pathogen detection or antibiotic management (Glimåker et al., 2020; Figueiredo et al., 2020) In our study, patients who died from CNSi were already critically ill upon admission, and some had progressed to an irreversible stage before receiving the test results, which may have limited the potential impact of advanced pathogen diagnostics on survival. Second, the limited number of critically ill patients in this study means that detecting a difference in mortality would require a larger sample size. Therefore, future research should involve larger, multicenter, prospective studies with more timely testing and optimized antimicrobial management to comprehensively evaluate the effect of mNGS on survival outcomes.

Recognizing that CNSi frequently represents a manifestation of systemic disease, often accompanied by complications such as sepsis, and pneumonia, or occurs as a complication following cranial surgery, (Lucas et al., 2013) we therefore conducted detailed subgroup analyses among patients with CNSi to identify populations deriving maximal benefit from the significant advantage of mNGS in accelerating clinical improvement compared to CMTs. Our results suggested that the statistically significant acceleration of clinical improvement associated with mNGS compared to CMTs was observed only in patients with concurrent pneumonia. No such difference was observed in patients without pneumonia comorbidity. Pathogen profiling within the mNGS group revealed that CNSi patients complicated with pneumonia harbored more frequently respiratory causative pathogens, such as Streptococcus pneumoniae, Klebsiella pneumoniae, and Acinetobacter baumannii, when compared to those free from pneumonia. These pathogens typically originate from nasopharyngeal colonization or medical devices, subsequently disseminating hematogenously and penetrating the blood-brain barrier, a pathogenic cascade associated with particularly adverse outcomes (Figueiredo et al., 2020; Mook-Kanamori et al., 2011) It is important to note that not all pathogens detected in the CSF are necessarily causative agents of CNSi; their presence could merely reflect the existence of nucleic acid fragments. Nevertheless, the detection of these pathogens indicates a high pathogen burden at the primary infection site, signifying more severe disease. (Chekrouni et al., 2024) Importantly, antibiotic therapy exerts systemic effects. Alleviating symptoms in both the CNS and other infectious foci contributes positively to the overall clinical outcome.

CNSi is one of the most common and devastating complications following neurosurgical surgery, which occurs in 1-7% of cases and is most often associated with prolonged treatment and significant morbidity. (Srinivas et al., 2011; Van Roy et al., 2024) In this study, clinical improvement was also more pronounced in post-neurosurgical patients within the mNGS group. However, the interaction effect was not statistically significant, likely due to the limited sample size. Regarding septic patients, the sample size in this subgroup was also limited. Although the clinical improvement rates between the mNGS and CMT groups did not reach statistical significance in the sepsis subgroup analysis, the limited sample size precludes definitive conclusions regarding the influence of sepsis. These findings underscore the necessity for prospective validation in larger cohorts to confirm mNGS’s role in optimizing the management of complex CNSi.

In routine clinical practice, empirical antimicrobial therapy is often initiated when CNSi is suspected, with subsequent adjustments guided by CSF inflammatory markers or pathogen detection results. However, such empiric regimens may introduce therapeutic bias and negatively impact patient outcomes (Campion and Scully, 2018; Ciummo et al., 2021) In our study, although all patients received empirical treatment before the availability of mNGS or CMTs, antimicrobial regimens were optimized in 24.3% of patients in the mNGS group. This advantage was most pronounced in patients with confirmed CNSi and those with concurrent pneumonia. Our results demonstrated that mNGS led to more frequent modifications of the antibiotic regimen than the CMT group in patients with CNSi concurrent with pneumonia. This indicated that the superior pathogen identification capability of mNGS effectively guided adjustments to antibiotic therapy, thus contributing to the acceleration of clinical improvement.

Our findings corroborate previous research establishing mNGS’s exceptional sensitivity in detecting both common pathogens in acute meningitis and fastidious or rare infectious agents. (Feng et al., 2020; Zou et al., 2022) mNGS consistently identified pathogens recalcitrant to CMTs, including parasites, ureaplasma species, Treponema pallidum, and Mycobacterium tuberculosis. Particularly, mNGS is advantageous for viral pathogen detection: when CSF mNGS yields exclusively viral-positive results, bacterial infection can be confidently excluded, supporting antimicrobial de-escalation. Although CSF routine examination is commonly used as the diagnostic basis for meningitis, previous studies have shown that some patients diagnosed with bacterial meningitis may have normal CSF cell counts (Fitch and van de Beek, 2007) In our study, CSF protein levels and CRP showed higher sensitivity in reflecting disease prognosis among patients with CNSi.

This study possesses several notable strengths. To our knowledge, it represents the first comparative assessment of the real-world clinical and therapeutic impact of mNGS versus CMT in CNSi, diagnostic criteria for meningitis, potential confounders, and outcome evaluation frameworks were defined *a priori* (Liu et al., 2025). Importantly, our findings provide preliminary evidence supporting the substantial potential of mNGS to improve clinical outcomes in CNSi patients, offering meaningful complementary evidence for its clinical value. Nevertheless, several limitations inherent to the retrospective design warrant acknowledgment. First, dynamic changes in mNGS and CMT results during treatment could not be systematically captured. Second, despite robust PSM, residual confounding may still affect the interpretation of clinical outcomes. Third, the relatively modest sample size-particularly after exclusions necessitated by incomplete records-constrains generalizability and limits statistical power for subgroup analyses, potentially introducing bias. Finally, the exclusion of patients with severe traumatic brain injury and indeterminate outcomes may restrict applicability to critically ill populations.

## 5 Conclusion

In this retrospective study, we found that integrating mNGS into the diagnostic pathway for suspected CNSi significantly accelerates clinical improvement compared to CMTs alone. The most substantial benefits were observed in patients with concurrent pneumonia high-risk subgroup frequently infected by aggressive respiratory pathogens. We posit that this clinical advantage stems from mNGS’s capacity for comprehensive and expeditious pathogen identification, which in turn enables earlier implementation of optimized, pathogen-directed antimicrobial therapy. These findings underscore the potential of mNGS to transform the management paradigm for complex CNSi, particularly in patients with systemic manifestations. Prospective, multicenter studies with larger cohorts are warranted to validate these observations and further define the patient populations deriving the greatest benefit from this advanced diagnostic modality.

## Data Availability

The data presented in the study are deposited in the National Center for Biotechnology Information (NCBI) repository, accession number PRJNA1344181.
