# Supplementary figures and images for: Impact of metagenomic sequencing on clinical outcomes in patients with suspected central nervous system infections: a retrospective case-control study
Source: Front Cell Infect Microbiol. 2025 Nov 7;15:1677092. doi: 10.3389/fcimb.2025.1677092 (PMC12634551; doi:10.3389/fcimb.2025.1677092)

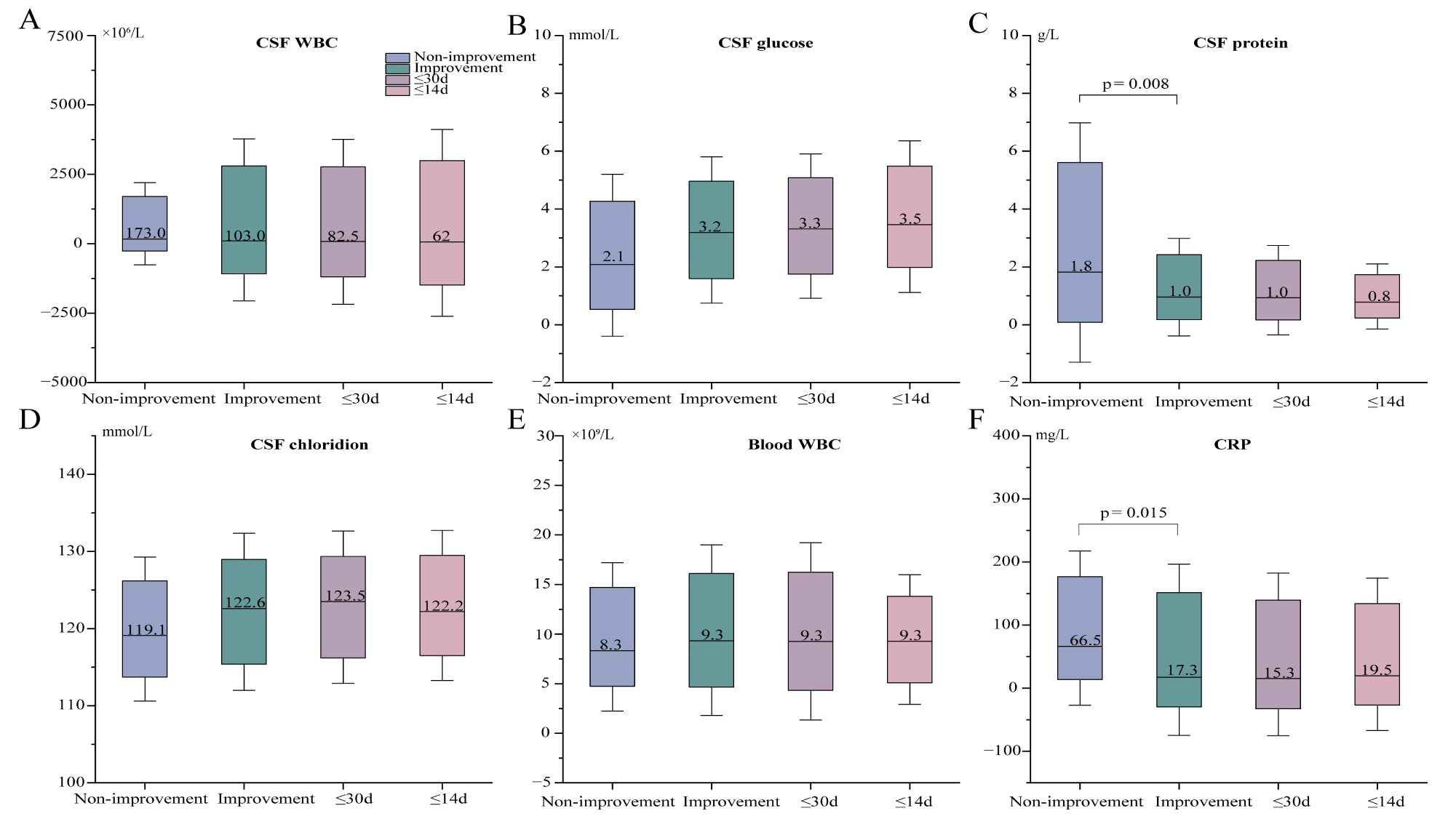

Supplement: Supplementary file 1 [file Image1.tif]
