# Supplementary material for: Impact of metagenomic sequencing on clinical outcomes in patients with suspected central nervous system infections: a retrospective case-control study
Source: Front Cell Infect Microbiol. 2025 Nov 7;15:1677092. doi: 10.3389/fcimb.2025.1677092 (PMC12634551; doi:10.3389/fcimb.2025.1677092)
Supplement: Supplementary file 2 [file Presentation1.pdf]

3Supplementary Material

1 Supplementary information

1.1 Supplementary information on the definition

1.1.1 Immunocompromised Status

Patients were defined as immunocompromised if they met at least one of the following criteria: Active malignancy, Primary immunodeficiency (e.g., HIV infection), Diabetes mellitus, Solid organ transplantation, Hematopoietic stem cell transplantation, Receipt of chemotherapy for malignancy, History of splenectomy, Long-term use of immunosuppressive agents.(Glimåker et al., 2020; Brouwer et al., 2010)

1.2 Supplementary information on Methods

1.2.1 mNGS workflow

1.2.1.1 CSF DNA/RNA extraction, library construction and sequencing

Pathogens′ detection by mNGS was performed as previously reported. (Sun et al., 2025; You et al., 2025)The microbial DNA was extracted from 600μL CSF using a QIAamp® UCP Pathogen DNA Kit (Qiagen, Valencia, CA, USA) following the manufacturer′s instructions. After nucleic acid extraction, the isolated DNA was quantified using a Qubit dsDNA HS Assay Kit (Invitrogen, USA),(Amar et al., 2021; Zhou et al., 2020)and then libraries were constructed using a Nextera XT DNA Library Prep Kit (Illumina, San Diego, CA). Total RNA from CSF samples was extracted using the QIAamp® Viral RNA Kit (Qiagen, Valencia, CA, USA), then subjected to human rRNA depletion by application of Ribo-Zero rRNA Removal Kit (Illumina, San Diego, CA, USA), following the manufacturer′s instructions. After removing the contaminating DNA via DNase I, cDNA was generated through reverse transcriptase and dNTPs. Nextera XT DNA Library Prep Kit (Illumina) was then applied for the construction of cDNA library. Library concentration was measured using a Qubit dsDNA HS Assay Kit, and library quality was evaluated with an Agilent 2100 Bioanalyzer (Agilent Technologies, Santa Clara, CA, USA) utilizing a High Sensitivity DNA kit. The libraries were sequenced on an Illumina NextSeq 550 sequencer for 75 cycles of single-end sequencing.(Miller et al., 2019)

1.2.1.2 Bioinformatic analysis

Trimmomatic was used to remove low quality reads, adapter contamination, and duplicate reads, as well as those shorter than 50 bp.(Bolger et al., 2014) Low complexity reads were removed by Komplexity with default parameters.(Clarke et al., 2019) Human sequence data were identified and excluded by mapping to a human reference genome (hg38) using Burrows-Wheeler Aligner software.(Li et al., 2009) Taxonomic profiles for all metagenomic samples were generated using Kraken 2 (v2.1.3)(Wood et al., 2019) and Bracken v2.5 (https://doi.org/10.7717/peerj-cs.104) with default settings. The database used for quantifying taxonomic profiles was constructed using a combined database containing human, bacterial, fungal, archaeal, and viral genomes downloaded from NCBI RefSeq (https://benlangmead.github.io/aws-indexes/k2).

To account for variations in sequencing depth across samples, we normalized the sequencing reads using reads per million (RPM). The optimal positive cutoff value for each species was determined by the parameter that yielded the highest area under the curve (AUC). For microorganisms without culture isolates, the RPM mean value and standard deviation of this microorganism were calculated, and the RPM (mean + 3SD) was set as a positive cutoff value.(Jing et al., 2021) The clinical reportable range (CRR) for pathogens was established according to the following three references described in a previous studies(Jing et al., 2021): Ⅰ. Johns Hopkins ABX Guide (https://www.hopkinsguides.com/hopkins/index/Johns_Hopkins_ABX_Guide/Pathogens), Ⅱ. Manual of Clinical Microbiology, and Ⅲ. clinical case reports or research articles published in peer

1.2.1.3 Control

To monitor potential sources of contamination, samples spiked with microorganisms were classified as positive samples, plasma from healthy donors was used as the negative control, and sterile deionized water was used as the Non-Template Control. Both controls were processed in parallel with other samples in each batch.(Miller et al., 2019; Grundy et al., 2023)In addition, sterile cotton swabs moistened with sterile deionized water were used to wipe the surfaces of the centrifuge and biosafety cabinet, generating a background microorganism profile for our laboratory.

1.2.2 Conventional microbiological test

The conventional microbiological tests (CMTs) encompassed bacterial and fungal culture and smear, India ink staining for Cryptococcus spp, culture and identification of Mycobacterium tuberculosis, nucleic acid amplification tests (NAATs) for cytomegalovirus (CMV), and Epstein-Barr virus (EBV), and serologic assays for herpesvirus antibodies.

2 References

1. Glimåker M, Naucler P, Sjölin J. (2020) Etiology, clinical presentation, outcome and the effect of initial management in immunocompromised patients with community acquired bacterial meningitis. *J Infect*;80(3):291–297.doi: 10.1016/j.jinf.2019.12.019.
2. Brouwer MC, Tunkel AR, van de Beek D. (2010) Epidemiology, diagnosis, and antimicrobial treatment of acute bacterial meningitis. *Clin Microbiol Rev*;23(3):467–492.doi: 10.1128/cmr.00070-09.
3. Sun Q, Teng R, Shi Q, Liu Y, Cai X, Yang B, et al. (2025) Clinical implement of Probe-Capture Metagenomics in sepsis patients: A multicentre and prospective study. *Clin Transl Med*;15(4):e70297.doi: 10.1002/ctm2.70297.
4. You H, Yang B, Liu H, Wu W, Yu F, Lin N, et al. (2025) Unravelling distinct patterns of metagenomic surveillance and respiratory microbiota between two P1 genotypes of Mycoplasma pneumoniae. *Emerg Microbes Infect*;14(1):2449087.doi: 10.1080/22221751.2024.2449087.
5. Amar Y, Lagkouvardos I, Silva RL, Ishola OA, Foesel BU, Kublik S, et al. (2021) Pre-digest of unprotected DNA by Benzonase improves the representation of living skin bacteria and efficiently depletes host DNA. *Microbiome*;9(1):123.doi: 10.1186/s40168-021-01067-0.
6. Zhou Z, Ren L, Zhang L, Zhong J, Xiao Y, Jia Z, et al. (2020) Heightened Innate Immune Responses in the Respiratory Tract of COVID-19 Patients. *Cell Host Microbe*;27(6):883–890.e882.doi: 10.1016/j.chom.2020.04.017.

7. Miller S, Naccache SN, Samayoa E, Messacar K, Arevalo S, Federman S, et al. (2019) Laboratory validation of a clinical metagenomic sequencing assay for pathogen detection in cerebrospinal fluid. *Genome Res*;29(5):831–842.doi: 10.1101/gr.238170.118.
8. Bolger AM, Lohse M, Usadel B. (2014) Trimmomatic: a flexible trimmer for Illumina sequence data. *Bioinformatics*;30(15):2114–2120.doi: 10.1093/bioinformatics/btu170.
9. Clarke EL, Taylor LJ, Zhao C, Connell A, Lee JJ, Fett B, et al. (2019) Sunbeam: an extensible pipeline for analyzing metagenomic sequencing experiments. *Microbiome*;7(1):46.doi: 10.1186/s40168-019-0658-x.
10. Li H, Durbin R. (2009) Fast and accurate short read alignment with Burrows-Wheeler transform. *Bioinformatics*;25(14):1754–1760.doi: 10.1093/bioinformatics/btp324.
11. Wood DE, Lu J, Langmead B. (2019) Improved metagenomic analysis with Kraken 2. *Genome Biol*;20(1):257.doi: 10.1186/s13059-019-1891-0.
12. Jing C, Chen H, Liang Y, Zhong Y, Wang Q, Li L, et al. (2021) Clinical Evaluation of an Improved Metagenomic Next-Generation Sequencing Test for the Diagnosis of Bloodstream Infections. *Clin Chem*;67(8):1133–1143.doi: 10.1093/clinchem/hvab061.
13. Grundy BS, Parikh H, Jacob S, Banura P, Moore CC, Liu J, et al. (2023) Pathogen Detection Using Metagenomic Next-Generation Sequencing of Plasma Samples from Patients with Sepsis in Uganda. *Microbiol Spectr*;11(1):e0431222.doi: 10.1128/spectrum.04312-22.

3 Supplementary Figures and Tables

3.1 Supplementary Table

Supplementary Table 1 Baseline characteristics of CNSi patients according to causative pathogens

	Bacterial(n=107)	Viral(n=70)	Mixed(n=20)	Unknown(n=29)	*p*
**Demographics**					
Age,median (IQR)	46.0(11.0-59.0)	35.0(9.5-59.3)	57.0(26.0-67.3)	48.0(12.0-56.0)	0.513
Male, n (%)	64.0(59.8%)	48.0(68.6%)	10.0(50.0%)	16.0(55.2%)	0.364
Onset time, median (IQR)	6.0(3.0-12.0)	6.0(3.3-10.0)	6.0(3.0-13.8)	4.0(2.0-8.0)	0.366
**Clinical symptoms, n(%)**					
Fever	93.0(86.9%)	53.0(75.7%)	15.0(75.0%)	20.0(69.0%)	0.088
Headache	56.0(52.3%)	42.0(60.0%)	9.0(45.0%)	21.0(72.4%)	0.160
Vomiting	39.0(36.4%)	21.0(30.0%)	7.0(35.0%)	10.0(34.5%)	0.851
Seizures	14.0(13.1%)	23.0(32.9%)	3.0(15.0%)	4.0(13.8%)	0.009
**Underlying, n (%)**					
Electrolyte	55.0(51.4%)	24.0(34.3%)	13.0(65.0%)	8.0(27.6%)	0.008
Hypoalbuminemia	34.0(31.8%)	11.0(15.7%)	7.0(35.0%)	5.0(17.2%)	0.051
Hypertension	34.0(31.8%)	6.0(8.6%)	7.0(35.0%)	9.0(31.0%)	0.002
Diabetes	25.0(23.4%)	8.0(11.4%)	3.0(15.0%)	1.0(3.4%)	0.035
**Risk factors, n (%)**					
Pneumonia	57.0(53.3%)	25.0(35.7%)	9.0(45.0%)	5.0(17.2%)	0.003
Sepsis	19.0(17.8%)	5.0(7.1%)	7.0(35.0%)	0(0%)	0.001
Immunocompromised	28.0(26.2%)	18.0(25.7%)	5.0(25.0%)	3.0(10.3%)	0.338
Antibiotic exposure	91.0(85.0%)	34.0(48.6%)	17.0(85.0%)	23.0(79.3%)	<0.001
**Invasive surgery, n (%))**					
IMV	19.0(17.8%)	4.0(5.7%)	2.0(10.0%)	3.0(10.3%)	0.115
Neurosurgical surgery	27.0(25.2%)	4.0(5.7%)	6.0(30.0%)	23.0(79.3%)	<0.001
**Laboratory examination, median (IQR)**					
CSF WBC, $\times 10^6$/L	302.0(72.5-1376.0)	55.5(20.5-189.5)	135.5(54.5-1481.5)	500.0(91.0-1266.0)	<0.001
CSF glucose, mmol/L	2.9(1.6-3.7)	3.5(2.8-4.0)	3.3(1.6-4.4)	3.0(2.0-4.4)	0.018
CSF protein, g/L	1.4(0.8-2.3)	0.7(0.4-1.0)	1.5(0.8-2.2)	1.3(0.7-2.3)	<0.001

CSF chloride, mmol/L	121.8(117.4-125.7)	122.5(119.3-125.2)	122.4(118.6-127.2)	123.5(119.7-129.0)	0.607

Due to the small number of CNSi caused by fungal and atypical pathogens (syphilis, tuberculosis, and Ureaplasma), these cases were not included in the table analysis, IMV : Invasive mechanical ventilation

Supplementary Table2 Post-hoc tests for significant differences in multiple comparisons

Variable	Pairwise Comparison	P for adjust	Direction of Effect
CSF WBC	Bacterial > Viral	< 0.001	Bacterial significantly higher than Viral
	Viral < Unknown	0.0006	Unknown significantly higher than Viral
CSF glucose	Bacterial < Viral	0.009	Viral significantly higher than Bacterial
CSF protein	Bacterial > Viral	< 0.001	Bacterial significantly higher than Viral
	Viral < Mix	0.007	Mix significantly higher than Viral
	Viral < Unknown	0.002	Unknown significantly higher than Viral
Antibiotic exposure	Viral < Bacterial	< 0.001	Viral group significantly lower than Bacterial
Neurosurgical surgery	Viral < Bacterial	0.011	Viral group significantly lower than Bacterial
	Unknown > Bacterial	< 0.001	Unknown significantly higher than Bacterial
	Unknown > Viral	< 0.001	Unknown significantly higher than Viral
	Unknown > Mix	0.012	Unknown significantly higher than Mix
Sepsis	Mix > Viral	0.047	Mix significantly higher than Viral
	Unknown < Mix	0.011	Unknown significantly lower than Mix
Hypertension	Viral < Bacterial	0.003	Viral significantly lower than Bacterial
Pneumonia	Unknown < Bacterial	0.007	Unknown significantly lower than Bacterial
Seizures	Viral > Bacterial	0.027	Viral significantly higher than Bacterial

3.2 Supplementary Figure

Supplementary Figure 1. Differences in laboratory indicators among CNSi patients with different clinical outcomes within the mNGS group.
